# Yogurt Consumption Is Associated with Lower Levels of Chronic Inflammation in the Framingham Offspring Study

**DOI:** 10.3390/nu13020506

**Published:** 2021-02-04

**Authors:** Mengjie Yuan, Martha R. Singer, Lynn L. Moore

**Affiliations:** Preventive Medicine and Epidemiology, Department of Medicine, Boston University School of Medicine, Boston, MA 02118, USA; mjyuan@bu.edu (M.Y.); msinger@bu.edu (M.R.S.)

**Keywords:** yogurt, dairy, chronic inflammation, biomarkers, prospective study, community dwelling participants

## Abstract

Some studies suggest that dairy foods may be linked with less chronic inflammation. However, few studies have investigated the separate effects of different types of dairy on inflammation. Therefore, the current study aims to examine the separate prospective impacts of milk, yogurt and cheese on biomarkers of chronic inflammation in 1753 community-dwelling participants of the Framingham Offspring Study (FOS). Mean intakes of dairy foods were derived from two sets of three-day diet records. Six inflammatory biomarkers were assessed approximately seven years later at exam 7. Results showed that those who consumed yogurt (vs. those who did not) had statistically significantly lower levels of interleukin-6 (IL-6) (mean log-transformed levels of 1.31 and 1.26 in consumers/non-consumers, respectively, *p* = 0.02) and fibrin (mean log-transformed levels of 5.91 and 5.89 in consumers/non-consumers, respectively, *p* = 0.03). The inverse association between IL-6 and yogurt consumption was similar in participants who were of normal weight and those who were overweight. For fibrin, the effects were stronger in overweight individuals. No statistically significant associations were observed between any of these inflammation biomarkers and milk or cheese intakes. Overall, our study compared the separate impacts of three types of dairy foods on chronic inflammation and found that only yogurt intake was linked with lower levels of chronic inflammation.

## 1. Introduction

Acute inflammation is a biological process in which the immune system responds to perceived harmful stimuli. However, systemic chronic inflammation, characterized by persistently high levels of circulating inflammatory biomarkers, contributes to diseases that collectively represent the leading causes of death globally, such as type 2 diabetes (T2DM), cancer and cardiovascular diseases (CVD) [1]. 

Diet plays an important role in modulating chronic inflammation. Prior research suggests that foods high in saturated fat (SFA) could promote chronic inflammation [2,3], although the dietary source of the SFA may be important [4]. Dairy foods are nutrient-dense and contain a variety of fatty acids, proteins, minerals, vitamin D, and other bioactive components. It has been suggested by some that the SFA content of dairy foods may initiate or promote an inflammatory response [5], but evidence on this topic is inconsistent [6]. Some clinical trials have found no impact of dairy foods intake on inflammation [7,8]. In contrast, others suggest that intakes of dairy foods, including high-fat dairy, may protect against systemic inflammation by suppressing inflammatory biomarkers such as C-reactive protein (CRP), interleukin-6 (IL-6) and tumor necrosis factor alpha (TNFα) in both healthy and obese subjects [9,10]. Such anti-inflammatory effects have been attributed to several nutrients, including calcium [11], magnesium [12], monounsaturated fatty acids (MUFA) [13], and probiotics [14] in dairy foods. However, dairy products include a range of different foods, from yogurt to cheese, and the profile of nutrients and bioactive compounds varies from food to food. For example, fermented dairy foods may have different effects on the inflammatory response than non-fermented foods [15,16]. In addition, many yogurts contain significant probiotics, which could alleviate inflammation by modulating gut microbiota, a key regulator of immunity [14]. 

To date, most studies addressing the impact of dairy intakes on inflammation have been short-term interventions focusing on dairy foods as a whole. Therefore, the current study aims to investigate the separate associations of milk, yogurt and cheese on markers of chronic inflammation in a prospective analysis of community-dwelling participants from the Framingham Offspring Study (FOS).

## 2. Method

### 2.1. Study Population

The National Heart, Lung, and Blood Institute’s Framingham Heart Study is a community-based, longitudinal study initiated in 1948 for the purpose of identifying factors that contribute to cardiovascular disease. In 1971, an offspring cohort was initiated and enrolled a sample of 5124 men and women who were the offspring of the original cohort and their spouses. Participants returned for examinations at approximately four-year intervals starting at exam 2 (1980). At each examination visit, participants underwent a series of clinical measurements, including a physical examination, anthropometry measures, medical history and laboratory assessments. 

Diet in the FOS included the use of three-day diet records during two examination cycles: exam 3 (1984–1988) and exam 5 (1991–1995). Inflammation-related biomarkers, including CRP, IL-6, TNFα, intercellular adhesion molecule (ICAM1), monocyte chemoattractant protein (MCP1) and fibrin, were measured at exam visit 7 (1998–2001). For these analyses, we used mean dietary intakes from examination cycles 3 and 5 as a measure of usual diet. Of the original 5124 participants, 3054 completed these three-day diet records. We excluded the following subjects at baseline: those with prevalent cancer (*n* = 98); BMI < 18.5 (*n* = 10); <30 years old (*n* = 6); extreme values for energy intakes (men: <1200 kcal or >4000 kcal, women: <1000 kcal or >3500 kcal, *n* = 176); extreme values for dairy food intakes (>6 s/d of milk or >6 s/d of yogurt or >4 s/d of cheese, *n* = 2); and missing potential confounding variables (*n* = 2). We also excluded those who were missing (*n* = 868); or had extreme levels for inflammatory biomarkers (i.e., CRP > 15 mg/L, IL-6 > 15 pg/mL, TNFα > 6 pg/mL, or ICAM1 > 500 ng/mL, *n* = 139). After these exclusions, 1753 subjects remained for the analysis.

### 2.2. Ethical Statement

The Framingham Studies are conducted in accordance with the Declaration of Helsinki guidelines and the protocol were conducted under the approval of Institutional Review Board of the Boston University Medical Center (IRB H-32132). All participants provided written informed consent. This study was registered at clinicaltrials.gov as NCT00005121.

### 2.3. Dietary Assessment

Diet was assessed using three-day diet records following standardized protocols with instructions from a trained nutritionist. Subjects were asked to record everything they ate and drank (including details on brands and recipes) on three consecutive days, including at least one weekend day. Mean intakes of milk, yogurt and cheese were then expressed as cup-equivalents (cup-eq) of dairy group based on the USDA’s MyPyramid definition of one cup-eq: milk (1 cup), yogurt (1 cup or 8 oz, while a typical yogurt container size ranges from 4–6 oz), and cheese (1.5 oz hard cheeses such as cheddar, mozzarella or parmesan; 2 oz American (processed) cheese, 1/3 cup shredded cheese, ½ cup ricotta cheese, and 2 cups cottage cheese). 

### 2.4. Inflammation Biomarker Assessment

Inflammatory biomarkers including CRP, IL-6, TNFα, ICAM1, MCP1, and fibrin were measured at exam 7. Fasting samples were obtained and stored at −80 °C. Standard quality control evaluations were performed, and all intra-assay coefficients of variation were <10%. CRP was measured using a high-sensitivity assay (Dade Behring Diagnostic, Deerfield, IL, USA). Other biomarkers were measured by commercially available enzyme-linked immunoassay kits: ICAM1 (R&D Systems, Minneapolis, MN, USA), IL-6 (R&D Systems, Minneapolis, MN, USA), MCP1 (R&D Systems, Minneapolis, MN, USA), and fibrin (Diagnostica Stago, Parsippany, NJ, USA). Values for individual biomarkers were log transformed before analysis after adding a constant of 1.0 to prevent negative values after log transformation.

### 2.5. Potential Confounders and Effect Modifiers

The following potential confounding variables were included in the final models: sex, age, body mass index (BMI, kg/m^2^), smoking status (current vs. non-current smoking), and the intakes of fruit and non-starchy vegetables. Other potential confounders, such as Health Eating Index (HEI) scores, education level, physical activity levels, use of nonsteroidal anti-inflammatory drugs, and the intake of meats and whole grain, were evaluated, but were not found to confound the relation between dairy foods and inflammation, and were, therefore, dropped from the final models. Finally, we also explored adjusting each dairy food group for other types of dairy foods (e.g., adjusting milk for yogurt and cheese intakes). Since none of these factors changed the effect estimates by more than 5%, they were not retained in the final models. Potential effect modification by BMI was also explored. 

### 2.6. Statistical Analysis

Sensitivity analyses were used to classify the mean intakes (from exams 3 and 5) of yogurt, milk and cheese from the three-day records as follows: yogurt (none vs. some), milk (≤1 vs. >1 cup-eq/d) and cheese (≤0.5 vs. >0.5 cup-eq/d). Generalized linear modeling was used to compare the log-transformed least squares mean values of individual inflammatory biomarkers according to different categories of intake of yogurt, milk and cheese. For the analysis of effect modification, subjects were dichotomized into two categories: normal BMI and overweight (BMI > 25 kg/m^2^ for females and BMI > 30 kg/m^2^ for males). Statistical analyses were carried out using SAS, version 9.4 (SAS Institute Inc., Cary, NC, USA).

## 3. Results

Table 1 describes the characteristics of subjects according to mean intakes of yogurt, milk and cheese at baseline. Yogurt consumers took in an average of 0.28 cup-eq per day (equivalent to approximately three 5.3-ounce containers of yogurt per week). Yogurt consumers were more likely to be female, were younger, and had lower BMIs than those who did not consume yogurt at all. They also consumed more fruit, non-starchy vegetables and whole grains than non-consumers of yogurt. In contrast, non-consumers were more likely to be current smokers, drank more alcohol and had a lower HEI score. In terms of milk intake, subjects who consumed more than 1 cup-eq per day tended to drink less alcohol, consumed less total energy and had a higher HEI compared with those who drank less milk. In contrast, participants consuming more cheese (>0.5 cup-eq/d) consumed more alcohol and had lower HEI scores than those consuming less cheese (≤0.5 cup-eq/d).

Table 2 shows the association between different dairy food intakes and log-transformed values for individual biomarkers after adjusting for age, sex, baseline BMI, smoking status, and intakes of fruit and non-starchy vegetables. Yogurt intake was associated with statistically significantly lower log-transformed values for IL-6 (mean ± SE in yogurt consumers vs. non-consumers: 1.26 ± 0.02 vs. 1.31 ± 0.01, *p*-value = 0.02) and Fibrin (mean ± SE: 5.89 ± 0.01 vs. 5.91 ± 0.01, respectively, *p*-value = 0.03). There were no associations between yogurt and other inflammation biomarkers and no statistically significant differences in any of these biomarkers associated with intakes of milk or cheese. 

To evaluate whether the effects of yogurt, milk or cheese on these pro-inflammatory biomarkers were modified by baseline BMI, we dichotomized subjects according to baseline BMI and then evaluated the effects of dairy food intakes (yogurt, milk, cheese) in stratified analyses in Table 3, Table 4 and Table 5, respectively. These tables show that overweight participants had higher levels of nearly all individual inflammation biomarkers, regardless of the dairy intake. While these stratified analyses have lower statistical power, there was a tendency for subjects who consumed yogurt to have lower levels of IL-6 regardless of BMI. Log-transformed Fibrin levels were also inversely associated with yogurt intake, particularly in normal-weight subjects. There was no association between yogurt intake and levels of CRP, TNF-α, ICAM1 or MCP1. 

Finally, we explored the associations between milk (Table 4) and cheese (Table 5) intakes and inflammatory biomarkers among normal weight and overweight subjects. There were no statistically significant or consistent associations observed in these analyses. 

## 4. Discussion

In the present study, we explored the effects of yogurt, milk and cheese intakes on biomarkers of chronic inflammation in a community-based population. We found that yogurt consumption was associated with lower levels of two inflammatory biomarkers: IL-6 and fibrin. In contrast, the intakes of milk and cheese had no statistically significant effects on any of the individual inflammation biomarkers. We also observed that the inverse association between yogurt intake and IL-6 was present in both normal weight and overweight individuals, although these effects had lower statistical power. For the subjects with normal baseline BMI, there was a slightly stronger inverse association between yogurt consumption and fibrin levels. Thus, we found that any beneficial effects of individual dairy foods in these analyses were limited to yogurt intake; furthermore, there was no consistent adverse effect of dairy foods on biomarkers of inflammation. To our knowledge, this is the first study to explore the long-term associations between separate dairy products intakes and biomarkers of inflammation. 

Yogurt is obtained by fermentation of milk with *Lactobacillus bulgaricus* and *Streptococcus thermophiles*, and some yogurt contains probiotics strains [17]. There is strong evidence that probiotics have anti-inflammatory properties both in animal models and in human studies [18,19]. One experiment reported that the administration of a yogurt containing lactic acid bacteria significantly suppressed the levels of pro-inflammatory cytokines, including TNFα, IL-6 and MCP1 in visceral adipose tissue and serum in obese mouse models [20]. Another study found that eight weeks of probiotic treatment led to a concurrent decrease of pro-inflammatory gene expression (TNFα, IL-6 and MCP1) in the adipose tissue of high-fat diet induced obese mice [21]. The anti-inflammatory capacities of probiotics have also been observed in humans. A recent meta-analysis of clinical trials suggested that probiotic treatments led to a reduction in pro-inflammatory biomarkers, including CRP and TNFα [22]. However, the anti-inflammatory impacts of probiotics are not consistent and differences may exist among strains [18].

The benefits of probiotics on ameliorating inflammation could be attributed to its capacity to modulate gut microbiota. A number of animal studies have suggested that gut microbiota play an important role in initiating chronic inflammation [23]. Microbiota dysbiosis leads to the disruption of intestinal epithelial barrier and induces lipopolysaccharide (LPS)-related endotoxemia, which in turn activates immune cells and triggers cytokine production [24]. Clinical trials have demonstrated that supplementation with probiotic yogurt attenuated endotoxemia by restoring the integrity of the intestinal epithelial barrier in older individuals with abnormal intestinal permeability [25]. Moreover, Zeng et al. compared the effects of yogurt vs. milk on reducing intestinal permeability in 30 irritable bowel syndrome patients and concluded that only yogurt treatment significantly improved mucosal barrier function [26]. Pei et al. found, in a nine-week clinical trial, that 339 g of yogurt per day significantly attenuated inflammation and inhibited endotoxin by improving intestinal barrier functions [27]. Therefore, the role of probiotics in regulating microbiota and reducing endotoxin exposure could explain some of the anti-inflammatory benefits of yogurt compared with other dairy foods. Although most cheeses are fermented, only some types of raw cheeses made from unpasteurized milk contain probiotics.

Even though increasing numbers of studies have indicated that yogurt reduces inflammation, it is not clear which inflammatory biomarkers it targets. Previous studies have shown that different inflammation biomarkers, such as CRP, TNFα and IL-6, were differently associated with body composition and these may further differ by obesity level and sex [28,29]. These findings may also explain the different effects of yogurt on different biomarkers. One clinical trial indicated that eight weeks of probiotic yogurt treatment significantly reduced the TNFα level, but while levels of IL-6 and CRP were also decreased, these findings were not statistically significant among those with diabetes [30]. Similarly, Pei et al. found that yogurt consumption significantly lowered TNFα levels but only resulted in a non-statistically significant decline in IL-6 levels among obese subjects [27]. In contrast, a recent meta-analysis with nine trials concluded that probiotic yogurt intakes lowered CRP levels, but had minimal effects on TNFα and IL-6 [31]. 

In the current study, we found significantly reduced IL-6 and fibrin levels in yogurt consumers, but failed to find associations with other inflammation biomarkers, including CRP, TNFα, ICAM1, and MCP1. Several factors may explain these discrepancies. First, most of the previous studies were short-term clinical trials with yogurt interventions ranging from 2 to 9 weeks; these short-term interventions with higher intakes of yogurt among previous non-yogurt consumers may well differ from those studying the effects of longer-term usual dietary patterns. Whether these effects will be sustained in the long run is largely unknown. The longer-term follow-up in the current study is of particular value, as it may be difficult to translate results from short-term trials to the understanding of longer-term effects of usual diet. Furthermore, the doses in these clinical trials are much higher than those seen in our cohort. Most of the studies administrated 200~400 g of yogurt daily in their experimental groups, while the average yogurt intake was 0.28 cup-eq (65 g) per day in our cohort. The relatively low yogurt intake as well as the small numbers of yogurt consumers during that period in the U.S. limits our ability to capture the benefits of higher yogurt consumption in this cohort. Another possible explanation for the differences between studies could relate to differences in the underlying metabolic health of the individuals being studied. IL-6 is a cytokine acting on the hepatocytes to induce the production of CRP and fibrin [32]. Approximately 35% of IL-6 is secreted by adipose tissue and the level of IL-6 is increased as BMI increases [33,34]. Therefore, it is logical that overweight individuals who consumed yogurt may have a somewhat stronger reduction in IL-6 compared with their normal weight counterparts. 

We observed significantly lower fibrin levels among yogurt consumers in this study. In accordance with our findings, one 12-week clinical trial found that both calcium and vitamin D-fortified Persian yogurt lowered plasma fibrin levels [35]. Fibrin is an acute-phase reactant, and its levels rise in response to inflammatory status and tissue injury. It plays an important role in thrombus formation and plasma viscosity, and has been identified as an independent risk factor for coronary heart disease and stroke [36]. The inverse association between yogurt intake and fibrin suggests that yogurt may provide preventive benefits for both cardiovascular disease and stroke. However, since studies examining the impacts of yogurt on fibrin are scarce, more studies are needed to confirm this important finding and to reveal the potential underlying mechanisms of this effect.

In the current study, we found that intakes of milk and cheese had no consistent effects on any of the six individual inflammation biomarkers. Since the majority of previous studies focused on the combined effects of dairy foods, the separate impacts of milk and other dairy foods on inflammation remain unclear. In a clinical trial, single meals containing low-fat milk led to lower levels of three inflammatory markers (IL-6, IL-1b and TNFα) after 3 hours (hrs) among overweight subjects [37]. However, in a four-week long study, Beavers et al. reported that daily low-fat milk consumption led to a non-statistically significant elevation in both IL-6 and TNFα levels in healthy postmenopausal women [38]. Moreover, one study found the expression of nuclear factor-kB (NF-kB), a key molecule initiating the inflammatory response, was significantly elevated 6 h after intake of whole milk in healthy subjects. The study also found a trend toward increasing ICAM1 and vascular cellular adhesion molecule 1 (VCAM1) levels associated with the whole milk treatment [39]. In contrast, a randomized trial among men found that whole milk did not significantly increase inflammatory response [40]. These conflicting results indicate that the association between milk intake and inflammation may differ based on the types of milk, the duration of treatment, or underlying subject characteristics. Since only a limited number of studies have focused on the anti-inflammatory effect of milk alone, the true association between milk consumption and inflammation is still unknown. Due to the high SFA content, cheese has been thought by some to promote inflammation. However, one clinical intervention found no significant difference in the CRP levels between subjects with regular-fat cheese treatment versus subjects with low-fat cheese treatment [41]. Furthermore, a 2017 review found that clinical trials investigating the relation between cheese intake and inflammatory biomarkers have had inconsistent results [6]. 

Some limitations of the current study must be acknowledged. First, more than 80% of the subjects in our study did not consume yogurt, and the average yogurt intakes of the consumer group were relatively low, which limits our ability to detect the benefits of higher levels of yogurt intake on chronic inflammation. Secondly, the inflammation biomarkers in this study were only collected at a single exam. Thirdly, diet was assessed at exams 3 and 5, leading to an average of 6.9 years between the measurement of usual yogurt intake and these inflammation biomarkers. However, several studies have shown that an adult’s diet tends to remain stable over time [42,43], suggesting that these measures of usual diet are likely to reflect yogurt intake more proximal to the outcome assessment as well. To the extent that this is not the case, these results are likely to underestimate the true effects of yogurt on inflammation. Furthermore, although we have excluded extremely high values of inflammatory biomarkers, it is still possible that the levels observed may still reflect acute inflammation, illness or injury rather than chronic subclinical levels of inflammation. Finally, we were unable to distinguish between conventional yogurt vs. probiotic yogurt in this study and, as a result, probiotics may not explain the anti-inflammatory effects of yogurt. 

## 5. Conclusions

In this study, we explored the usual intakes of yogurt, milk and cheese in relation to several inflammatory biomarkers with an average of approximately seven years of follow up, and found that yogurt consumption was associated with lower levels of both IL-6 and fibrin in these healthy middle-aged and older individuals. These results suggest that yogurt consumption may be an important part of a healthy diet, designed to mitigate systemic inflammation.

## Figures and Tables

**Table 1:** Baseline subject characteristics according to category of dairy food intake.

	Yogurt				Milk				Cheese			
	None		Some		≤1 Cup-eq/d		>1 Cup-eq/d		≤0.5 Cup-eq/d		>0.5 Cup-eq/d	
	*N* = 1428		*N* = 325		*N* = 1185		*N* = 568		*N* = 1149		*N* = 604	
	Mean	SD	Mean	SD	Mean	SD	Mean	SD	Mean	SD	Mean	SD
Age (yrs)	55.86	9.59	53.92	8.70	55.55	9.21	55.39	9.96	56.53	9.41	53.54	9.24
BMI (kg/m^2^)	26.98	4.44	26.27	4.31	26.93	4.49	26.66	4.28	26.77	4.49	26.98	4.28
Physical Activity (Mets)	14.71	7.96	14.63	7.85	14.63	7.98	14.82	7.84	14.61	7.68	14.85	8.40
Alcohol (g/d)	9.46	13.17	7.54	10.35	10.12	13.63	6.98	10.25	8.11	11.90	11.00	13.95
Energy intake (kcals/d)	1920	534	1855	523	1814	503	2104	539	1806	498	2103	542
HEI 2015 Score	56.16	11.14	62.55	11.17	56.58	11.17	58.96	11.75	58.92	11.44	54.37	10.76
Yogurt (cup-eq/d)	0.00	0.00	0.28	0.27	0.05	0.16	0.05	0.16	0.05	0.17	0.05	0.12
Milk (cup-eq/d)	0.89	0.75	0.83	0.67	0.47	0.26	1.72	0.70	0.89	0.75	0.86	0.72
Cheese (cup-eq/d)	0.44	0.40	0.45	0.42	0.44	0.40	0.43	0.42	0.21	0.15	0.88	0.38
Meat (oz-eq/d)	2.59	1.63	1.88	1.34	2.43	1.60	2.52	1.62	2.32	1.54	2.72	1.69
Poultry (oz-eq/d)	1.43	1.28	1.47	1.24	1.45	1.30	1.42	1.22	1.49	1.27	1.34	1.27
Fish (oz-eq/d)	1.23	1.20	1.29	1.17	1.26	1.21	1.20	1.17	1.32	1.23	1.09	1.11
Eggs (per day)	0.39	0.36	0.32	0.32	0.38	0.36	0.38	0.34	0.36	0.34	0.42	0.37
Nuts, Seeds (oz-eq/d)	0.48	0.79	0.55	0.87	0.47	0.81	0.54	0.79	0.45	0.75	0.59	0.89
Whole Grains (oz-eq/d)	0.62	0.70	0.83	0.88	0.59	0.72	0.80	0.77	0.66	0.70	0.66	0.81
Fruit, Non-starchy Vegetables (cup-eq/d)	2.54	1.37	3.06	1.46	2.61	1.38	2.68	1.45	2.65	1.45	2.59	1.31
	*N*	%	*N*	%	*N*	%	*N*	%	*N*	%	*N*	%
Sex (female)	714	50.0	239	73.5	683	57.6	270	47.5	671	58.4	282	46.7
Current smoker	224	15.7	23	7.1	173	14.6	74	13.0	157	13.7	90	14.9

Abbreviations: BMI, body mass index; cup-eq/d, cup-equivalent per day; g/d, gram per day; HEI, Healthy Eating Index; kcals, kilocalories; Mets, metabolic equivalents; oz-eq, oz-equivalent per day; SD, standard deviation; yrs, years.

**Table 2:** Adjusted mean levels of inflammation biomarkers according to dairy food intake category.

Biomarkers ^1^	Yogurt			Milk			Cheese		
	None	Some		≤1 Cup-eq/d	>1 Cup-eq/d		≤0.5 Cup-eq/d	>0.5 Cup-eq/d	
	Mean ^2^ ± SE		*p*-Value	Mean ^2^ ± SE		*p*-Value	Mean ^2^ ± SE		*p*-Value
**Log-CRP**	1.19 ± 0.02	1.16 ± 0.03	0.40	1.18 ± 0.02	1.20 ± 0.02	0.51	1.19 ± 0.02	1.18 ± 0.02	0.86
**Log-IL-6**	1.31 ± 0.01	1.26 ± 0.02	0.02	1.29 ± 0.01	1.33 ± 0.02	0.09	1.31 ± 0.01	1.30 ± 0.02	0.67
**Log-TNFα**	0.81 ± 0.01	0.82 ± 0.02	0.84	0.81 ± 0.01	0.82 ± 0.01	0.69	0.81 ± 0.01	0.82 ± 0.01	0.54
**Log-ICAM1**	5.50 ± 0.01	5.48 ± 0.01	0.26	5.50 ± 0.01	5.49 ± 0.01	0.47	5.49 ± 0.01	5.49 ± 0.01	0.68
**Log-MCP1**	5.72 ± 0.01	5.74 ± 0.02	0.51	5.72 ± 0.01	5.74 ± 0.01	0.38	5.73 ± 0.01	5.72 ± 0.01	0.71
**Log-Fibrin**	5.91 ± 0.01	5.89 ± 0.01	0.03	5.91 ± 0.01	5.90 ± 0.01	0.24	5.90 ± 0.01	5.91 ± 0.01	0.70

Abbreviations: CRP, C-reactive protein; cup/d, cup per day; cup-eq/d, cup-equivalent per day; ICAM1, soluble intracellular adhesion molecule 1; IL-6, interleukin-6; MCP1, monocyte chemoattractant protein 1; TNFα, tumor necrosis factor alpha. ^1^ All biomarkers are log-transformed. ^2^ Least squares means are adjusted for age, sex, BMI, smoking status, fruit and non-starchy vegetables.

**Table 3:** Adjusted mean levels of inflammation biomarkers according to combined categories of yogurt intake and weight status.

Biomarkers ^1^	Weight Status ^2^	Yogurt Intake		
		None	Some	
		Mean ^3^ ± SE		*p*-Value
**Log-CRP**	Normal	1.05 ± 0.02	0.99 ± 0.04	0.20
	Overweight	1.42 ± 0.03	1.44 ± 0.05	0.78
**Log-IL-6**	Normal	1.24 ± 0.01	1.19 ± 0.03	0.11
	Overweight	1.43 ± 0.02	1.36 ± 0.04	0.08
**Log-TNFα**	Normal	0.80 ± 0.01	0.81 ± 0.02	0.70
	Overweight	0.83 ± 0.01	0.83 ± 0.03	0.86
**Log-ICAM1**	Normal	5.47 ± 0.01	5.45 ± 0.02	0.24
	Overweight	5.53 ± 0.01	5.52 ± 0.02	0.73
**Log-MCP1**	Normal	5.70 ± 0.01	5.72 ± 0.02	0.52
	Overweight	5.75 ± 0.01	5.76 ± 0.03	0.83
**Log-Fibrin**	Normal	5.88 ± 0.01	5.85 ± 0.01	0.03
	Overweight	5.96 ± 0.01	5.94 ± 0.01	0.41

Abbreviations: CRP, C-reactive protein; ICAM1, soluble intracellular adhesion molecule 1; IL-6, interleukin-6; MCP1, monocyte chemoattractant protein 1; TNFα, tumor necrosis factor alpha.^1^ All biomarkers are log-transformed. ^2^ Weight status is classified as normal weight or overweight (defined as BMI > 25 for females and BMI > 30 for males). ^3^ Least squares means are adjusted for age, sex, smoking status, fruit and non-starchy vegetables.

**Table 4:** Adjusted mean levels of inflammation biomarkers according to combined categories of milk intake and weight status.

Biomarkers ^1^	Weight Status ^2^	Milk Intake		
		≤1 Cup-eq/d	>1 Cup-eq/d	
		Mean ^3^ ± SE		*p*-Value
**Log-CRP**	Normal	1.04 ± 0.02	1.03 ± 0.03	0.88
	Overweight	1.41 ± 0.03	1.47 ± 0.04	0.28
**Log-IL-6**	Normal	1.22 ± 0.01	1.25 ± 0.02	0.23
	Overweight	1.41 ± 0.02	1.45 ± 0.03	0.25
**Log-TNFα**	Normal	0.80 ± 0.01	0.81 ± 0.02	0.46
	Overweight	0.83 ± 0.01	0.83 ± 0.02	0.73
**Log-ICAM1**	Normal	5.47 ± 0.01	5.47 ± 0.01	0.93
	Overweight	5.53 ± 0.01	5.51 ± 0.02	0.25
**Log-MCP1**	Normal	5.70 ± 0.01	5.71 ± 0.02	0.65
	Overweight	5.75 ± 0.02	5.77 ± 0.02	0.45
**Log-Fibrin**	Normal	5.88 ± 0.01	5.87 ± 0.01	0.13
	Overweight	5.96 ± 0.01	5.95 ± 0.01	0.92

Abbreviations: CRP, C-reactive protein; cup-eq/d, cup-equivalent per day; ICAM1, soluble intracellular adhesion molecule 1; IL-6, interleukin-6; MCP1, monocyte chemoattractant protein 1; TNFα, tumor necrosis factor alpha. ^1^ All biomarkers are log-transformed. ^2^ Weight status is classified as normal weight or overweight (defined as BMI > 25 for females and BMI > 30 for males); ^3^ Least squares means are adjusted for age, sex, smoking status, fruit and non-starchy vegetables.

**Table 5:** Adjusted mean levels of inflammation biomarkers according to combined categories of cheese intake and weight status.

Biomarkers ^1^	Weight Status ^2^	Cheese Intake		
		≤0.5 Cup-eq/d	>0.5 Cup-eq/d	
		Mean ^3^ ± SE		*p*-Value
**Log-CRP**	Normal	1.03 ± 0.02	1.04 ± 0.03	0.84
	Overweight	1.43 ± 0.03	1.42 ± 0.04	0.84
**Log-IL-6**	Normal	1.23 ± 0.02	1.24 ± 0.02	0.48
	Overweight	1.43 ± 0.02	1.39 ± 0.03	0.16
**Log-TNFα**	Normal	0.80 ± 0.01	0.82 ± 0.01	0.26
	Overweight	0.83 ± 0.01	0.83 ± 0.02	0.71
**Log-ICAM1**	Normal	5.47 ± 0.01	5.47 ± 0.01	0.91
	Overweight	5.53 ± 0.01	5.52 ± 0.01	0.69
**Log-MCP1**	Normal	5.70 ± 0.01	5.71 ± 0.02	0.85
	Overweight	5.76 ± 0.02	5.74 ± 0.02	0.44
**Log-Fibrin**	Normal	5.88 ± 0.01	5.87 ± 0.01	0.85
	Overweight	5.95 ± 0.01	5.97 ± 0.01	0.22

Abbreviations: CRP, C-reactive protein; cup-eq/d, cup-equivalent per day; ICAM1, soluble intracellular adhesion molecule 1; IL-6, interleukin-6; MCP1, monocyte chemoattractant protein 1; TNFα, tumor necrosis factor alpha. ^1^ All biomarkers are log-transformed. ^2^ Weight status is classified as normal weight or overweight (defined as BMI > 25 for females and BMI > 30 for males). ^3^ Least squares means are adjusted for age, sex, smoking status, fruit and non-starchy vegetables.

## Data Availability

Restrictions apply to the availability of these data. Data was obtained from the Framingham Heart Study and can be requested at https://framinghamheartstudy.org/fhsforresearchers/data-available-overview/.
